# A Comprehensive Review of the Effects of Extracorporeal Shock Wave Therapy on Stroke Patients: Balance, Pain, Spasticity

**DOI:** 10.3390/medicina59050857

**Published:** 2023-04-28

**Authors:** Jung-Ho Lee, Eun-Ja Kim

**Affiliations:** Department of Physical Therapy, Kyungdong University, 815, Gyeonhwon-ro, Munmak-eup, Wonju-si 26495, Gang-won-do, Republic of Korea; ljhcivapt@naver.com

**Keywords:** stroke, ESWT, balance, pain, spasticity

## Abstract

Stroke remains a leading cause of disability worldwide, with survivors often experiencing impairments in balance, pain, spasticity, and control that limit their ability to perform daily living activities. Extracorporeal shock wave therapy (ESWT) has emerged as a potential treatment modality to improve these outcomes in stroke patients. This review aims to provide a comprehensive examination of the effects of ESWT on stroke patients, focusing on the theoretical background, balance, pain reduction, muscle spasticity and control, and upper and lower extremities. This study reviewed the use of ESWT in treating balance, pain, and spasticity in stroke patients, focusing on articles published in PubMed between January 2003 and January 2023. Systematic reviews related to stroke were used to provide an overview of stroke, and a total of 33 articles related to balance, pain, and spasticity were selected. ESWT has several shock wave generation methods and application methods, and it has been shown to have positive therapeutic effects on various aspects of rehabilitation for stroke patients, such as improving balance, reducing pain, decreasing muscle spasticity and increasing control, and enhancing functional activities of the upper and lower extremities. The efficacy of ESWT may vary depending on the patient’s condition, application method, and treatment area. Therefore, it is important to apply ESWT according to the individual characteristics of each patient in clinical practice to maximize its potential benefits.

## 1. Introduction

Stroke is a leading cause of disability and death worldwide. It is estimated that over 17 million people suffer a stroke each year, with many experiencing long-lasting impairments in their physical and cognitive abilities [1]. Stroke is a complex medical condition that occurs when blood flow to the brain is interrupted or reduced, leading to brain cell damage or death [2]. The most common type of stroke is ischemic stroke, which occurs when a blood clot blocks a blood vessel in the brain [3]. This can occur either in a large artery (such as the middle cerebral artery) or in a smaller blood vessel within the brain. As a result of the blockage, blood flow to a specific part of the brain is disrupted, and the brain tissue in that area does not receive enough oxygen and nutrients. This causes the brain cells to begin to die within minutes, leading to irreversible brain damage. Another type of stroke is hemorrhagic stroke, which occurs when a blood vessel in the brain ruptures, causing bleeding in or around the brain. The bleeding can put pressure on the surrounding brain tissue, leading to brain damage or death [4].

The risk factors for stroke include hypertension, high cholesterol, smoking, diabetes, obesity, family history of stroke, and age [5]. These risk factors can cause damage to the blood vessels in the brain, making them more susceptible to blockage or rupture. Prevention is key in reducing the impact of stroke. Managing risk factors such as hypertension, high cholesterol, and smoking can help to reduce the risk of stroke [6]. The pathogenesis of stroke also involves a series of inflammatory responses and chemical changes within the brain tissue [7]. After the initial injury, immune cells are activated and begin to release cytokines, chemokines, and other inflammatory mediators. These mediators cause further damage to the brain tissue, leading to a cascade of events that can exacerbate the injury [8].

The consequences of stroke can be devastating, with survivors often facing significant challenges in their daily lives. Physical impairments resulting from stroke may include weakness, numbness, or paralysis of one side of the body. This can make it difficult to perform everyday activities such as dressing, grooming, and eating. Stroke survivors may also experience difficulties with balance and coordination, making it harder to walk or stand without assistance [9]. Cognitive impairments resulting from stroke may include difficulty with memory, attention, and language. This can make it difficult to communicate with others, remember important information, and perform tasks that require concentration and problem-solving skills [10].

The long-term effects of stroke can also lead to reduced independence and quality of life. Many stroke survivors require ongoing support and care from family members or healthcare professionals, which can place a significant burden on their loved ones and on the healthcare system. Early identification and treatment of stroke can also improve outcomes and reduce the severity of disability. Rehabilitation is an important aspect of recovery for stroke survivors, and may include physical therapy, occupational therapy, and speech therapy. These therapies can help to improve physical and cognitive abilities, and enable stroke survivors to regain independence and improve their quality of life [11].

Extracorporeal shock wave therapy involves the use of shock waves to stimulate tissue repair and regeneration, reduce pain, and improve function [12]. There are four methods used to generate shock waves in ESWT: electrohydraulic, electromagnetic, piezoelectric, and radial pressure wave. The electrohydraulic method uses an electrode to generate a spark that produces a shock wave in water, which is transmitted to the patient. The electromagnetic method uses a coil to generate a strong magnetic field that accelerates a projectile, generating a shock wave when it strikes a metal applicator. Piezoelectricity uses a piezoelectric crystal to create shock waves when an electric current is applied. The radial pressure wave method uses a projectile that is accelerated by compressed air to generate a radial pressure wave when it strikes a metal applicator. The energy level and penetration depth of the shock wave depend on the method used and can be adjusted by changing the electrical or air pressure parameters, and the size and shape of the projectile or crystal [13,14,15,16].

Focused extracorporeal shock wave therapy (F-ESWT) is a type of ESWT that uses a highly focused shock wave to treat specific areas of the body [17,18]. F-ESWT is typically used to treat musculoskeletal conditions such as tendinitis [19], plantar fasciitis [20], and bone fractures [21]. F-ESWT has several advantages over other types of ESWT. It is highly precise and accurate, allowing for targeted treatment of specific areas. The high-energy shock wave can penetrate deep into the tissue, making it suitable for treating deep tissue injuries. F-ESWT also has a relatively short treatment time and requires minimal recovery time [22]. However, F-ESWT also has some potential risks and side effects. These can include pain or discomfort during the procedure, bruising or swelling at the treatment site, and rare but serious complications such as nerve damage or infection [23].

Radial extracorporeal shock wave therapy (R-ESWT) is a type of ESWT that uses a radial shock wave to treat various musculoskeletal conditions. This type of therapy is used for treating chronic tendinopathies [24], lateral epicondylitis [25], plantar fasciitis [26], and other soft tissue injuries [27]. In R-ESWT, a projectile is accelerated by compressed air, striking the surface of the applicator, which generates radial waves that spread out over a wider area of tissue. The energy level of the radial shock wave can be adjusted depending on the specific condition being treated, and the number of pulses delivered can vary as well [22]. The device delivers radial waves of energy into the tissues, stimulating the healing process by increasing blood flow and promoting tissue regeneration. One of the main advantages of R-ESWT is that it is a non-invasive and low-risk procedure, making it a safer alternative to other types of treatments such as surgery or steroid injections. It is also a relatively quick procedure, with each session lasting only a few minutes. However, R-ESWT may not be as effective as focused ESWT for treating deep tissue injuries [28]. In recent years, extracorporeal shock wave therapy (ESWT) has gained attention as a potential treatment for post-stroke complications. ESWT involves the application of shock waves that can stimulate tissue repair and regeneration, reduce pain, and improve function [12,13]. This review aims to synthesize the current evidence on the effects of ESWT in stroke patients, with a focus on theoretical background, balance, pain reduction, balance improvement, muscle spasticity and control, and upper and lower extremities.

## 2. Materials and Methods

To conduct a comprehensive literature review on the potential use of ESWT in treating stroke patients’ symptoms of balance, pain, and spasticity, the author utilized the PubMed platform and searched for articles using the terms “Stroke” and “ESWT” in combination with keywords such as “Balance”, “Pain”, and “Spasticity”. The review included clinical studies, systematic reviews, meta-analyses, pilot studies, and case reports published from January 2003 to January 2023 to ensure a comprehensive overview of the research on this topic. The final selection of articles for the review included only those published in English.

**Figure 1:**
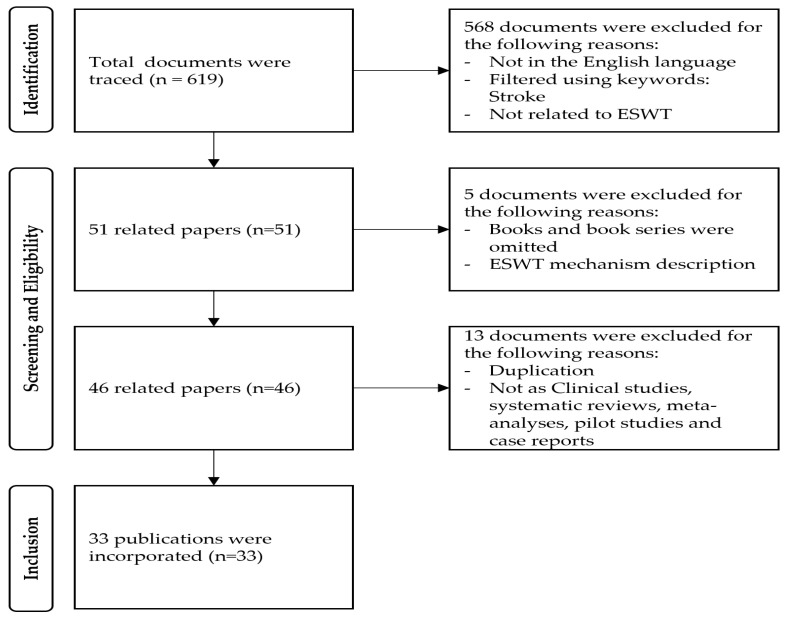
PRISMA flowchart.

The search yielded a significant number of potential sources of information, including 466 articles related to stroke and balance, 423 articles related to stroke and pain, and 161 articles related to stroke and spasticity using systematic review as a keyword. Additionally, the search found 39 articles related to stroke and ESWT, 15 articles related to ESWT and balance, 563 articles related to ESWT and pain, and 48 articles related to ESWT and spasticity. In addition to ensuring that the final selection of articles included only the most relevant and reliable sources of information on the potential use of ESWT in treating stroke patients’ symptoms of balance, pain, and spasticity, the authors also made sure to include studies that investigated the effectiveness of ESWT when used in combination with other treatments (Figure 1).

In order to write a comprehensive review paper, only systemic review papers on balance, pain, and spasticity related to stroke were found and used as data for an overall explanation of stroke. During the process of selecting papers for the review on the potential use of ESWT in treating balance, pain, and spasticity in stroke patients, the author excluded any papers that were not related to ESWT from the initial search results for papers related to ESWT and balance or pain or spasticity as keywords. Following the exclusion of any papers unrelated to ESWT, the author selected 13 articles related to balance, 17 articles related to pain, and 21 articles related to spasticity in stroke patients. After checking for any duplication, a total of 33 articles were finally selected for the review. After the final articles were selected, the author carefully read the full text and evaluated the content. This rigorous selection process ensured that only the most relevant and reliable sources of information on the potential use of ESWT in treating balance, pain, and spasticity in stroke patients were included in the review paper.

The exclusion criteria for the review on the use of ESWT in stroke patients were as follows: (1) Studies in which ESWT was applied but stroke patients were not the subjects were excluded. (2) Studies that did not provide a clear explanation of the application method of ESWT were excluded. (3) Studies that only described the method and mechanism of ESWT were excluded.

## 3. Effects of ESWT on Balance Improvement in Stroke

Balance impairments are common following a stroke, often resulting from a combination of factors, including motor weakness, sensory deficits, impaired coordination, and reduced cognitive function [29]. Damage to the motor cortex, cerebellum, or brainstem can disrupt the neural pathways responsible for maintaining balance and coordinating movement. Additionally, muscle weakness or spasticity in the affected limbs can further compromise stability during standing or walking. As a result, stroke survivors may experience an increased risk of falls, reduced mobility, and diminished independence in daily activities [30,31].

ESWT has been proposed as a potential treatment for balance impairments in stroke patients due to its ability to stimulate tissue repair and regeneration, reduce pain, and improve muscle function [32]. ESWT may help improve balance by targeting the affected muscles and neural pathways, promoting neuromuscular re-education, and facilitating motor recovery [33]. The mechanical stimulation induced by ESWT can increase blood flow [28], promote cellular responses, and enhance the functionality of affected muscles and joints, thereby improving overall balance and stability [34]. Several clinical trials and systematic reviews have investigated the effects of ESWT on balance (Table 1) [35]. These studies have generally reported positive outcomes, with improvements in balance and postural control following ESWT treatment. For example, a clinical trial by Xiao et al. aimed to investigate the effects of whole-body vibration combined with extracorporeal shock wave therapy on spasticity and gait function in hemiplegic stroke patients [36]. The combined group had greater improvements in all measured outcomes compared to the control group. These findings suggest that whole-body vibration combined with ESWT can effectively improve spasticity and motor function in hemiplegic stroke patients. Another study, a randomized controlled trial, found that visual feedback balance training combined with radial extracorporeal shock wave therapy and conventional physiotherapy significantly improved lower limb spasticity, trunk control, and static and dynamic balance in post-stroke patients compared to a control group receiving visual feedback training and sham R-ESWT [37]. These results suggest that ESWT has the potential to effectively improve outcomes in the population of post-stroke patients with lower limb spasticity and balance impairments.

The efficacy of ESWT on outcomes in stroke patients can be influenced by several factors, including the type of ESWT [38], treatment parameters [39,40], and patient characteristics [41]. In a meta-analysis article [40], it was found that the selected studies did not explicitly mention the use of identical ESWT parameters. However, through meta-regression and subgroup analysis, it was shown that factors such as the number of shocks and the application site did not significantly impact the treatment’s effectiveness. This highlights the need for further research to determine the optimal shock wave intensity, frequency, and number parameters for reducing spasticity, suggesting that parameter variations may exist across the included studies. Additionally, the optimal ESWT protocol for balance improvement in stroke patients remains to be established, with further research needed to determine the most effective treatment parameters and strategies. In summary, ESWT has shown promising results in improving balance and postural control in stroke patients. However, further research is needed to optimize treatment protocols and identify the patient populations who may benefit most from this therapy.

## 4. Effects of ESWT on Pain Reduction in Stroke

Post-stroke pain can result from various factors, including direct neural injury, muscle spasticity, joint stiffness, or secondary musculoskeletal complications [42]. The most common type of pain, which is caused by damage to the central nervous system, is shoulder pain due to hemiplegic shoulder subluxation or spasticity [43]. Post-stroke pain can significantly impact a patient’s quality of life and hinder rehabilitation efforts [44]. The electromagnetic and Piezoelectric ESWT has been proposed as a potential treatment for pain reduction in patients due to its analgesic [45,46] and anti-inflammatory effects [5]. ESWT may alleviate pain by stimulating the release of endogenous pain-relieving substances, disrupting pain signaling pathways, and reducing local inflammation.

Clinical trials have shown promising results for ESWT in reducing pain in stroke patients (Table 2). The previously study examined the efficacy of ESWT for plantar fasciitis in stroke patients [47]. ESWT was found to decrease tension in the plantar fascia, leading to reduced pain and improved gait ability, with greater improvement observed after 6 months of treatment compared to after 6 weeks of treatment.

The systematic review aimed to assess the effects of extracorporeal shock wave therapy for shoulder pain after stroke [45]. The meta-analysis showed that ESWT had a significant positive effect on pain levels, motor function, active mobility, and comprehensive function of the shoulder compared to conventional treatment, as measured by visual analogue scale scores, Fugl-Meyer assessment upper extremity scale scores, active range of motion assessment, and functional comprehensive assessment scores. The study suggests that ESWT can improve pain levels, motor function, active mobility, comprehensive function of the shoulder, and activities of daily living in patients with shoulder pain after stroke. Another meta-analysis study evaluated the long-term effects of ESWT on spasticity after stroke and found that ESWT significantly reduced pain and spasticity as measured using a visual analog scale (VAS) and modified Ashworth scale (MAS), respectively [48]. It also improved joint range of motion and motor function as measured by FMA, with long-term effects observed in stroke patients.

## 5. Effects of ESWT on Muscle Spasticity and Control in Stroke

Stroke can cause muscle spasticity and control impairments due to damage to the motor cortex or other neural structures responsible for muscle control. This can result in impaired muscle activation, reduced motor unit recruitment, and altered muscle fiber composition, leading to decreased muscle strength and endurance [31]. ESWT may help improve muscle strength and endurance in stroke patients by enhancing blood flow, promoting tissue repair, and stimulating neuromuscular re-education. The mechanical stimulation induced by ESWT can also increase the production of growth factors [49] and cytokines [50], which may contribute to muscle repair and regeneration. Clinical trials and systematic reviews investigating the effects of ESWT on muscle spasticity and control in stroke patients have shown promising results (Table 3). All five studies in the table found positive effects of ESWT on spasticity in post-stroke patients, despite differences in the methodologies and sample sizes. Yang et al. [13] and Opara et al. [29] conducted narrative reviews and reported mixed results for functional recovery, while Guo et al. [32], Mihai et al. [31], and Jia et al. [48] performed meta-analyses or systematic reviews and found improvements in various parameters, such as the MAS, pain intensity, and range of motion. However, all studies emphasized the need for further research to optimize treatment parameters and better understand the underlying mechanisms. They also suggested that more large-scale, well-designed randomized controlled trials are needed to confirm the efficacy of ESWT in treating post-stroke spasticity and establish standardized protocols. Another study examined the impact of radial extracorporeal shock wave therapy on spasticity in stroke patients; the researchers found that three sessions of ESWT significantly reduced hand and wrist spasticity for at least 16 weeks, while also improving hand function and wrist control [51]. This suggests that ESWT may be valuable for stroke patients experiencing spasticity.

In a systematic review, the effectiveness of ESWT, including the focused (F-ESWT) and radial (R-ESWT) types, on post-stroke muscle spasticity and motor recovery was examined [12]. A total of 17 articles with 303 patients were reviewed, and the results indicated that both F-ESWT and R-ESWT led to subjective and objective improvements in post-stroke spasticity, pain, functioning, range of motion, postural control, muscular endurance, muscle tone, and muscle elasticity. In addition, slightly better effects of R-ESWT were observed in spasticity reduction and alpha motor neuron excitability, while F-ESWT showed more beneficial effects in range of motion improvement. Overall, the studies support the effectiveness of ESWT in reducing muscle spasticity and enhancing motor recovery after stroke. In a review paper investigating the impact of shock wave therapy on spasticity in stroke patients [39], researchers conducted a systematic analysis of existing studies to determine the effectiveness of this treatment. The review found that both focused and radial extracorporeal shock wave therapies led to significant improvements in spasticity, pain, functioning, range of motion, postural control, muscular endurance, muscle tone, and muscle elasticity. The studies collectively support the use of shock wave therapy as an effective intervention for reducing spasticity and enhancing motor recovery in stroke patients.

## 6. Effects of ESWT on Upper and Lower Extremities in Stroke

Upper and lower extremity impairments following stroke can result from a combination of motor, sensory, and cognitive deficits. The damage to neural structures responsible for motor control can lead to muscle weakness, impaired coordination, and reduced movement precision [5,8,11]. Additionally, sensory deficits may affect proprioception and tactile feedback, further compromising motor function [52]. ESWT may help improve upper and lower extremity function in stroke patients by stimulating tissue repair, promoting neuromuscular re-education, and facilitating motor recovery (Table 4) [39,41,51].

Among the papers related to the upper limb in stroke patients, one systematic review examined the effectiveness of ESWT for reducing spasticity and improving upper limb functionality in stroke survivors; 16 randomized controlled trials involving 764 individuals were analyzed, demonstrating that ESWT is effective in reducing upper limb spasticity and provides additional benefits for upper limb motor-function improvement in the short and medium term when combined with conventional therapy [53]. In a different study examining the effects of R-ESWT on flexor spasticity of the upper limb after stroke, a prospective, randomized, double-blind controlled trial with 100 participants aims to explore the mechanism of R-ESWT’s impact on spasticity [54]. By comparing active R-ESWT with sham-placebo R-ESWT using various assessments, this study may provide support for further investigation of the potential mechanisms and alternative treatment options for post-stroke spasticity management. In a previous study, the effect of R-ESWT on upper limb spasticity and functionality in chronic stroke patients was evaluated. When combined with conventional rehabilitation, R-ESWT significantly reduced wrist and hand spasticity and led to improvements in wrist control, hand function, and pinch grip strength, suggesting that R-ESWT may be an effective addition to conventional rehabilitation for stroke patients with upper limb spasticity.

Another systematic review aimed to evaluate the impact of ESWT on reducing lower extremity spasticity in adult stroke survivors [41]. ESWT was found to have a positive impact on spasticity, ankle range of motion, and lower extremity function in adult stroke survivors based on a systematic review of 12 studies with 278 participants. Additionally, ESWT was deemed a safe and effective non-invasive rehabilitation strategy for chronic stroke survivors, with no reported side effects. The authors suggest that ESWT could be a useful approach in improving gait for individuals who have suffered a stroke.

## 7. Future Directions and Challenges

The majority of studies have focused on the use of ESWT to reduce pain [45] in different fields. Furthermore, there have been very few studies exploring the potential of ESWT to improve balance in stroke patients. As such, there is a need for further research to explore the potential use of ESWT in treating stroke patients’ symptoms of balance and spasticity.

One of the critical areas for future research in ESWT for stroke rehabilitation is determining the optimal treatment protocols. This includes identifying the most effective type of ESWT (focused or radial) [17,18,19,21], treatment parameters (intensity, frequency, and number of sessions) [26], and the appropriate timing [40] for intervention. Further research should investigate these factors to establish evidence-based guidelines for ESWT application in stroke rehabilitation. Future research should also explore the most effective ways to integrate ESWT into comprehensive stroke rehabilitation programs [13,32]. This may involve evaluating the combined effects of ESWT with other therapeutic interventions [19,25,36], such as physical therapy, occupational therapy, and cognitive rehabilitation. Identifying the optimal combination and sequence of interventions could help maximize the benefits of ESWT and enhance overall stroke recovery.

**Figure 2:**
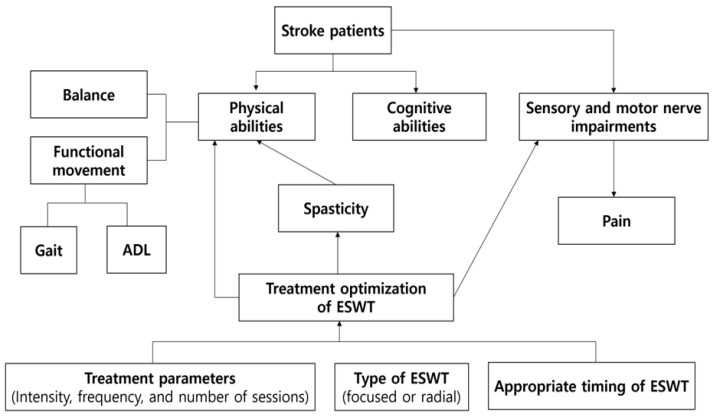
Decision-making Steps for Optimizing ESWT Applied to Treat Disability in Stroke Patients and the Impact on Injury Reduction. Abbreviations: ADL: activities of daily living; ESWT: extracorporeal shock wave therapy.

Personalized medicine has become increasingly important in healthcare, and ESWT interventions for stroke patients should be no exception. Future research should focus on identifying patient characteristics and individual factors that may influence the response to ESWT. This could include factors such as age, stroke severity, time since stroke, and comorbidities. Personalized ESWT interventions tailored to each patient’s unique needs and characteristics may lead to improved outcomes and a more efficient use of healthcare resources. Technological advancements in ESWT delivery may offer new opportunities for improving stroke rehabilitation outcomes [46]. For example, the development of portable, user-friendly ESWT devices [16] could enable greater access to treatment in various settings, including outpatient clinics, home-based care, and telemedicine. Additionally, advances in ESWT technology may lead to more precise targeting of affected muscles and neural pathways, potentially enhancing treatment efficacy (Figure 2).

Despite the promising findings in this review, several challenges and limitations must be addressed to fully understand the potential of ESWT in stroke rehabilitation. These include the heterogeneity of study populations and interventions, small sample sizes, and the lack of standardized outcome measures. Addressing these issues in future research will help to build a stronger evidence base for ESWT in stroke patients and provide valuable insights into its clinical implementation. Ultimately, the continued investigation and refinement of ESWT as a therapeutic modality could contribute to improved rehabilitation outcomes and quality of life for stroke survivors.

## 8. Conclusions

Based on the reviewed studies, it can be concluded that ESWT shows promise as a potential therapeutic intervention for stroke rehabilitation. ESWT has been shown to be effective in reducing pain, improving muscle spasticity, and enhancing upper and lower extremity function in stroke patients. The mechanical stimulation induced by ESWT promotes tissue repair, neuromuscular re-education, and motor recovery. However, there is a need for further research to optimize treatment parameters, identify patient characteristics that influence the response to ESWT, and integrate ESWT into comprehensive stroke rehabilitation programs. Personalized ESWT interventions and technological advancements in ESWT delivery may offer new opportunities for improving stroke rehabilitation outcomes. Addressing the challenges and limitations in current studies will help to build a stronger evidence base for ESWT in stroke rehabilitation and provide valuable insights into its clinical implementation. Overall, the continued investigation and refinement of ESWT as a therapeutic modality could contribute to improved rehabilitation outcomes and quality of life for stroke survivors.

## 9. Limitation of Study

This study warrants caution in interpreting its content due to the presence of numerous limitations. The primary limitation of this research is that the selection and exclusion process of the papers may be susceptible to errors, as there were only two authors involved in conducting the study. Such errors could have an impact on the outcomes and interpretations of the research. Another limitation of this study is the use of only one database for the literature search, which may have resulted in difficulties generalizing our findings and potentially providing incomplete or inaccurate information.

## Figures and Tables

**Table 1:** Clinical articles on the efficacy of extracorporeal shock wave therapy on balance.

Author	Method	Patients	Results	Conclusion
Bilek and Tekin (2021) [34]	ESWT + Neuro-Developmental Treatment (NDT) on children with unilateral cerebral palsy	32 children with unilateral cerebral palsy (CP)	Improved trunk control skills, PBS scores, and TUG test performances	ESWT combined with NDT improves postural control and balance in children with unilateral CP
Mihai et al. (2021) [35]	R-ESWT, visual feedback balance training, and conventional physiotherapy on lower limb post-stroke spasticity, trunk control, and balance	23 patients with lower limb post-stroke	Statistically significant improvements in clinical and stabilometric outcome measures, including balance, trunk performance, and limb function; reduced spasticity, pain intensity, and clonus	R-ESWT, visual feedback training, and conventional physiotherapy improve balance, trunk performance, sensorimotor outcomes, and limb function, and reduce spasticity, pain, and clonus in post-stroke patients
Xiao et al. (2022) [36]	WBV + ESWT on spasticity and gait in hemiplegic patients with stroke	50 hemiplegic patients with stroke	Improved spasticity, motor function, balance, and gait	WBV combined with ESWT improves spasticity, motor function, balance, and gait in hemiplegic patients with stroke

Abbreviations: ESWT: extracorporeal shock wave therapy; PBS: pediatric balance scale; TUG: timed up and go test; R-ESWT: radial-ESWT; WBV: whole body vibration.

**Table 2:** Clinical articles on the efficacy of extracorporeal shock wave therapy on pain in stroke patients.

Author	Method	Patients	Results	Conclusion
Taheri et al. (2017) [39]	ESWT 1 session/week for 3 weeks, oral anti-spastic medications, stretching exercises	28 hemiplegic stroke patients Group1: 14 patients (ESWT+drug+Ex.) Group2: 14 patients (Drug+Ex.)	Significant improvement in MAS, pain of ankle plantar flexor, ROM, 3m walk duration, and LEFS after ESWT treatment	ESWT combined with medications and exercises improved spasticity in stroke patients
Sohn et al. (2011) [11]	One session of focused ESWT on the medial head of the gastrocnemius	10 healthy adults 10 stroke patients	Significant reduction in MAS of plantar flexor Mild pain of ankle plantar flexor was experienced during ESWT	ESWT effectively improved ankle plantar flexor spasticity without impacting F wave or H-reflex parameters and did not cause an increase in pain for the patients
Kim et al. (2013) [30]	Radial ESWT (R-ESWT) every 2–3 days for 2 weeks on spastic subscapularis muscle	57 stroke patients	Reduction in MAS and VAS, improvement in ROM of shoulder Effects lasted up to 4 weeks after the last treatment	R-ESWT effectively and safely reduced spasticity and pain, improved ROM in spastic shoulders of stroke patients

Abbreviations: MAS: modified Ashworth scale; ESWT: extracorporeal shock wave therapy; Ex: exercise; ROM: range of motion; LEFS: lower extremity functional score; VAS: visual analog scale.

**Table 3:** Reviews of the efficacy of extracorporeal shock wave therapy on spasticity in stroke patients.

Author	Method	No. Studies/ No. Patients	Results	Conclusion/Limitation
Yang et al. (2021) [13]	Narrative review	8 RCTs/413 post-stroke patients	Positive effects on parameters such as the MAS Mixed results reported functional recovery	ESWT shows promise in treating spasticity; however, more research is needed to establish optimal parameters and to compare with other treatments
Guo et al. (2017) [32]	Meta-analysis	6 studies consisting of 9 groups (total patients not specified)	MAS grades were improved immediately after ESWT and at 4 weeks after ESWT	ESWT is effective in reducing spasticity in post-stroke patients, but more large-scale, well-designed RCTs are needed for confirmation
Opara et al. (2021) [29]	Narrative review	22 studies/468 post-stroke patients	ESWT reduces muscle tone in stroke ESWT is safe and free of undesirable side effects	Further research is needed to establish uniform muscle stimulation parameters using ESWT and to understand its mechanism of action
Mihai et al. (2020) [31]	Systematic review and meta-analysis	7 RCTs/170 post-stroke patients	ESWT ameliorates spasticity, reduces pain intensity, and increases ROM No side effects of ESWT	ESWT has long-term efficacy on lower limb post-stroke spasticity with a satisfactory safety profile
Jia et al. (2020) [48]	Meta-analysis of RCTs	8 RCTs/301 post-stroke patients	Significant reduction in MAS (WMD = −0.36) and VAS (WMD = −0.94) Enhanced ROM (WMD = 5.97) and FMA (WMD = 1.26)	ESWT demonstrated long-term effects in relieving spasticity, reducing pain, enhancing ROM and motor function in stroke patients

Abbreviations: RCTs: randomized controlled trials; MAS: modified Ashworth scale; ESWT: extracorporeal shock wave therapy; ROM: range of motion; VAS: visual analog scale; WMD: weighted mean difference.

**Table 4:** Clinical trials and reviews of the efficacy of extracorporeal shock wave therapy on upper and lower extremities in stroke.

Author	Method	No. Studies/ No. Patients	Results	Conclusion/Limitation
Cabanas-Valdés et al. (2020) [41]	Systematic review and meta-analysis	12 studies/278 participants	ESWT was effective in reducing lower limb spasticity and improving ankle range of motion and lower limb function in chronic stroke survivors	ESWT (radial/focused) is a safe and effective non-invasive rehabilitation strategy for reducing lower limb spasticity in chronic stroke survivors
Li et al. (2016) [51]	Randomized, single-blind, controlled trial	60 chronic stroke patients	R-ESWT was effective in reducing spasticity of the hand and wrist, and the reduction in spasticity after three sessions of R-ESWT was maintained for up to 16 weeks	R-ESWT may be valuable in decreasing spasticity of the hand and wrist with accompanying enhancement of wrist control and hand function in chronic stroke patients
Cabanas-Valdés et al. (2020) [53]	Systematic review and meta-analysis	16 randomized controlled trials/764 individuals	ESWT was effective in reducing upper limb spasticity, and adding it to conventional therapy provided an additional benefit	ESWT is effective for reducing upper limb spasticity and provides additional benefits when added to conventional therapy

Abbreviations: ESWT: extracorporeal shock wave therapy; R-ESWT: radial ESWT.

## Data Availability

Not applicable.
